# Using DNA metabarcoding as a novel approach for analysis of platypus diet

**DOI:** 10.1038/s41598-022-06023-y

**Published:** 2022-02-10

**Authors:** Tahneal Hawke, Gilad Bino, Michael E. Shackleton, Alexandra K. Ross, Richard T. Kingsford

**Affiliations:** 1 Centre for Ecosystem Science, School of Biological, Earth & Environmental Sciences, UNSW, Sydney, NSW 2052 Australia; 2 Centre for Freshwater Ecosystems (CFE), La Trobe University, University Drive, Wodonga, VIC 3690 Australia; 3 Evolution and Ecology Research Centre, School of Biological, Earth and Environmental Sciences, UNSW, Sydney, NSW Australia

**Keywords:** Ecology, Freshwater ecology

## Abstract

Platypuses (*Ornithorhynchus anatinus*) forage for macroinvertebrate prey exclusively in freshwater habitats. Because food material in their faeces is well digested and mostly unidentifiable, previous dietary studies have relied on cheek pouch assessments and stable isotope analysis. Given DNA metabarcoding can identify species composition from only fragments of genetic material, we investigated its effectiveness in analysing the diet of platypuses, and to assess variation across seasons and sexes. Of the 18 orders and 60 families identified, Ephemeroptera and Diptera were the most prevalent orders, detected in 100% of samples, followed by Trichoptera, Pulmonata, and Odonata (86.21% of samples). Caenidae and Chironomidae were the most common families. Diptera had a high average DNA read, suggesting it is an important dietary component that may have been underestimated in previous studies. We found no variation in diet between sexes and only minimal changes between seasons. DNA metabarcoding proved to be a highly useful tool for assessing platypus diet, improving prey identification compared to cheek pouch analysis, which can underestimate soft-bodied organisms, and stable isotope analysis which cannot distinguish all taxa isotopically. This will be a useful tool for investigating how platypus prey diversity is impacted by habitat degradation as a result of anthropogenic stressors.

## Introduction

Dietary analysis is important in determining habitat, resource, and metabolic requirements of species^1–3^. Changes in diet may track changing environmental conditions, highlighting vulnerability to future climate pressures^4,5^. It also gives insight into competitive interactions and predator–prey relationships^6,7^ and can provide information about the carrying capacity of particular habitats^8,9^. Understanding these requirements is critical for effective conservation management, including captive breeding programs^10^ which are likely to become increasingly important for declining species.

Mostly, traditional dietary analyses rely on visual observations, morphological identification of prey components in the stomach or faeces, or stable isotope analyses^11^. More recently, DNA-based methods are used because they can identify morphologically indistinct material, allowing greater precision in diet studies with more taxa detected^12–14^. Specifically, DNA metabarcoding can determine species’ composition by sequencing DNA from soft-bodied and hard-bodied taxa^15,16^. DNA metabarcoding has been used successfully to assess the diet of different mammal^13,17–19^ and fish species^14,20,21^.

Platypuses (*Ornithorhynchus anatinus*) are semi-aquatic monotremes, endemic to freshwater rivers and streams throughout eastern Australia^22^. They predominantly feed at night^22,23^ in water bodies^22^, using electroreception and mechanoreception in their bills to detect prey^24^. They forage for macroinvertebrates under submerged logs and rocks, under river banks, and by sifting through fine sediment^25^. Platypuses store freshly captured and partially processed food in their cheek pouches, evolved to replace the stomach’s function as a food storage organ^26^.

Given the nocturnal and aquatic nature of platypuses, visual assessments of diet are ineffective^11^. Additionally, due to the small stomach and mostly unidentifiable food material in the faeces^27^, dietary analyses have relied primarily on collecting and analysing cheek pouch samples^25,28–30^, and more recently stable isotope analysis^27^. Both methods have biases; mastication of prey from cheek pouches prevents morphological identification of some prey and stable isotope analysis cannot distinguish some taxa^27^.

Platypuses prey on a wide variety of benthic macroinvertebrates such as insects, crustaceans, worms, and molluscs, with the orders of Trichoptera, Ephemeroptera, Odonata, and Coleoptera most commonly consumed^25,27–30^. Their diet has been shown to vary seasonally in the wild^25,28^ and captivity^31,32^, with no reported differences between males and females.

We used DNA metabarcoding for the first time on platypus check pouch samples, collected from the Snowy and Upper Murray Rivers regions, to determine its viability for assessing the species’ diet. We predicted that this would be a successful approach in assessing platypus diet, while also potentially resulting in the detection of non-dominant orders and additional orders compared to those detected through morphological cheek pouch analysis. We also compared seasonal differences in diet between summer (breeding season, September–February) and autumn (non-breeding season, March–August) and between sexes. We predicted some variation between seasons and potentially between sexes in response to differences in metabolic demands between males and females and variable prey abundance throughout the year.

## Methods

### Sample collection

Cheek pouch samples were collected from platypuses captured during surveys^33^ using unweighted mesh nets or fyke nets. Mesh nets were set parallel to riverbanks in large, deep pools (> 50 m long, 1–2 m deep), from dusk until 1.00 A.M. and checked every few minutes with a spotlight in additional to a physical examination at least once an hour to remove possible snags. Platypuses were removed from the nets immediately upon being observed. Fyke nets were set in pairs in small shallow streams (< 1 m) in the late afternoon and checked every three hours until shortly after sunrise. Captured platypuses were transferred to pillowcases before processing, following established protocols^33,34^. Individuals were anesthetized in an induction chamber using isoflurane (Pharmachem, 5%) in oxygen (3 L/min), over 5–7 min^35,36^. Anaesthesia was maintained using a T-piece face mask, with isoflurane (1–1.5%) in oxygen (1.0L/min)^37^. Macroinvertebrate samples were collected from platypuses by inserting a small stainless-steel spoon into the buccal cavity via the bill^28^. The spoon was sterilised between individuals using 100% ethanol. Samples were stored at minus 80 °C until DNA extraction in October 2019. Trapping and handling of platypuses was carried out in accordance with guidelines and approved by the NSW Office of Environment and Heritage (SL101655), NSW Department of Primary Industries (P15/0096-1.0 & OUT15/26392), and UNSW’s Animal Care and Ethics Committee (16/14A), and in compliance with the ARRIVE guidelines.

**Figure 1:**
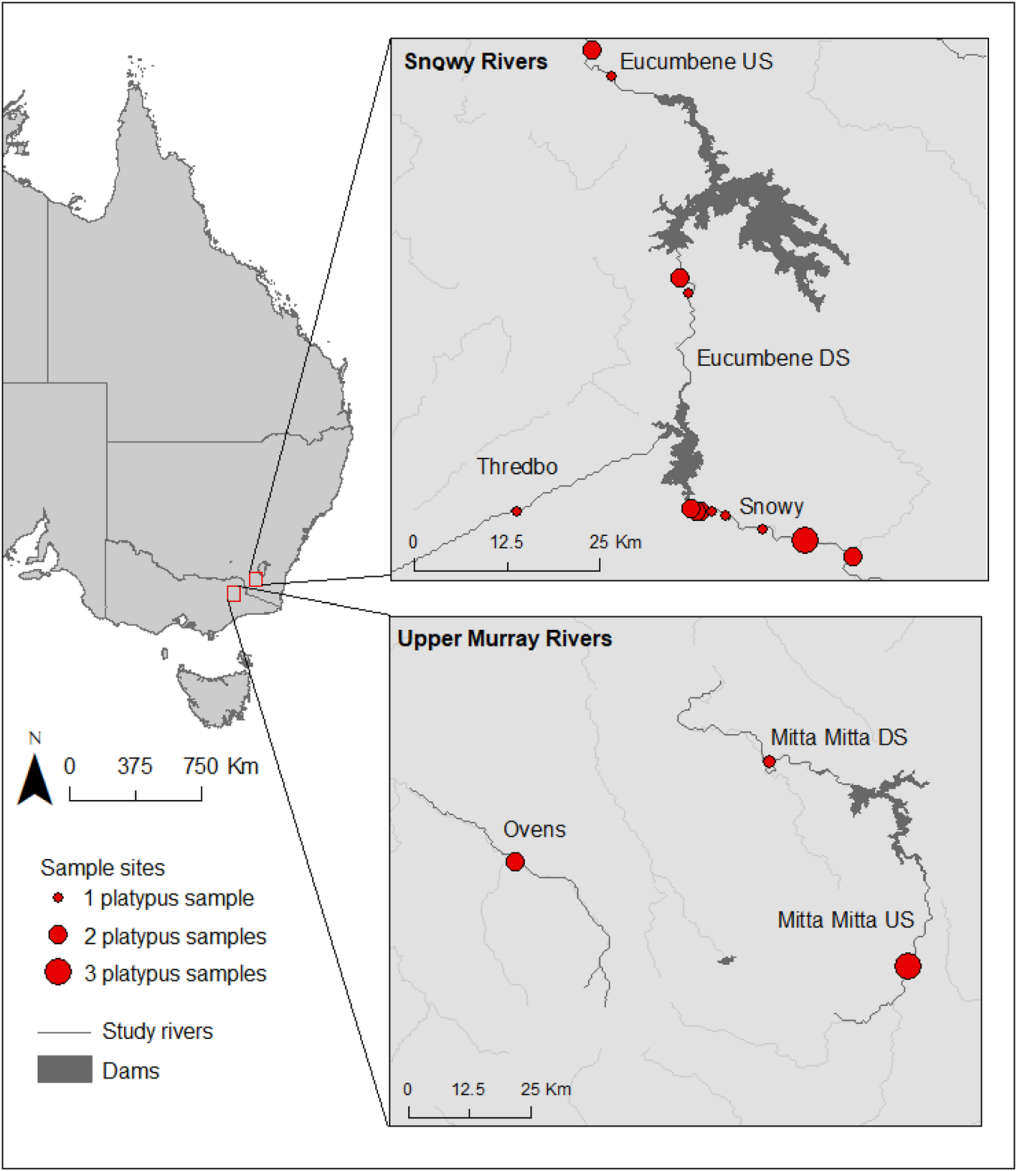
Sites along seven river sections (Snowy, Thredbo Eucumbene US and DS, Mitta Mitta US and DS, and Ovens) in the Snowy and Upper Murray Rivers where cheek pouch samples were collected from platypuses for dietary analysis (detailed description of sampled individuals and capture dates in Appendix 1) (figure was created with ArcGIS Desktop (Version 10.8) https://www.esri.com).

We analysed cheek pouch samples from 29 platypuses, collected between December 2016 and May 2018 from seven river sections within the Snowy Rivers and Upper Murray Rivers regions (Fig. 1). Within the Snowy River region, 23 samples were collected from the Thredbo, Snowy, and Eucumbene Rivers and six samples were collected from the Mitta Mitta and Ovens River in the Upper Murray Rivers region.

### DNA sequencing and analysis

DNA was extracted from platypus cheek pouch samples (approx. 0.05 cm^3^ of sample used after homogenisation) using the DNeasy Blood and Tissue Kit (QIAGEN, Hilden, Germany), following instructions from the manufacturer. All PCR and sequencing steps were performed by Mr DNA (Molecular Research, Shallowater, TX, USA; http://www.mrdnalab.com). A 313 base pair (bp) region of the COI gene was amplified using the primers mlCOIintF (GGWACWGGWTGAACWGTWTAYCCYCC) and jgHCO2198 (TAIACYTCIGGRTGICCRAARAAYCA)^38,39^. Unique index barcodes of 8 bp were attached to each primer. One-step PCRs were done in duplicate, using the HotStarTaq Plus Master Mix Kit (Qiagen, USA). Thermocycling protocols consisted of 94 °C for 3 min; 30 cycles of 94 °C for 30 s, 53 °C for 40 s and 72 °C for 1 min; and a final elongation step at 72 °C for 5 min. Successful amplification was checked on a 2% agarose gel. Samples were pooled in equal proportions, based on molecular weight, purified using Ampure XP beads and an Illumina DNA library prepared. Samples were sequenced on an Illumina MiSeq sequencer, using V2 300 cycle kit and PhiX spike-in was added at 20%.

### Taxonomic assignment

Sequence data from each sample was demultiplexed using custom-built FASTq Processor script (http://www.mrdnafreesoftware.com/). The Greenfield Hybrid Analysis Pipeline (GHAP)^40^ was used for processing data and creating operational taxonomic units (OTU) tables. Sequences with a minimum overlap of 25 bp and homology of at least 80% were merged and only sequences between 301 and 313 bp (i.e. close to the target amplicon length) were retained for further analyses^41^. Sequences were clustered into OTUs, using a 97% clustering threshold as this threshold corresponds reasonably well with COI delimitation between invertebrate species^42^. To remove possible sequencing artifacts, OTUs that made up less than 0.01% of the total reads in a sample were filtered out.

OTUs were associated with taxa by taking the highest percent homology matches from a reference library of global sequences from GenBank^43^ (https://www.ncbi.nlm.nih.gov/genbank/, accessed 10th of June 2021). We then refined this taxonomic assignment using percent homology thresholds, arbitrarily assigned as part of the GHAP pipeline: 97% or greater for species, 95 to < 97% for genus, 90 to  < 95% for family, 85- < 90% for order, and those with < 85% were not assigned because of taxonomic uncertainty^41^.

Freshwater snails (families Ancylini, Agriolimacidae, Lymnaeidae, and Physidae) were all assigned to the informal group (previously an order) Pulmonata. This informal group has been included as an order in the analysis for ease of interpretation.

### Analyses

To evaluate variation in diet between samples, seasons, and sex, we quantified four metrics for each taxon (order or family, depending on the taxonomic scale of analysis): (1) taxon diversity (average number of taxa in each sample); (2) taxon prevalence (the percentage of samples that each taxon was detected in); (3) taxon DNA reads (the average DNA read numbers of each taxon across all samples); (4) taxon relative prevalence (the proportion of samples that each taxon was detected in, relative to the total number of taxa detected for that pool of samples (total, season or sex)).

Due to the low number of samples collected during spring (n = 3) and winter (n = 2), we grouped samples from spring with summer, representing the breeding season (September–February) and samples from winter with autumn, representing the non-breeding season (March–August). Given the small sample sizes, and the nature of count and proportional data, we compared differences in the number of orders and families present in each sample between seasons and between sexes using non-parametric Wilcoxon Signed‐Rank tests^44^. We also compared differences in individual taxon DNA reads between seasons and sexes using Wilcoxon Signed-Rank tests. We plotted taxon accumulative curves using the ‘rarefy’ function in the ‘vegan’ package (v2.5-7)^45^. All analyses were implemented within R (v4.0.2)^46^.

## Results

### Metabarcoding effectiveness

A total of 2,346,944 reads were generated from the MiSeq run. Prior to analysis, several kingdoms, phyla, and orders were categorised as environmental DNA and removed from the analysis (Appendix 2). After data filtering, there were 1,389,491 reads attributable to invertebrates.

**Figure 2:**
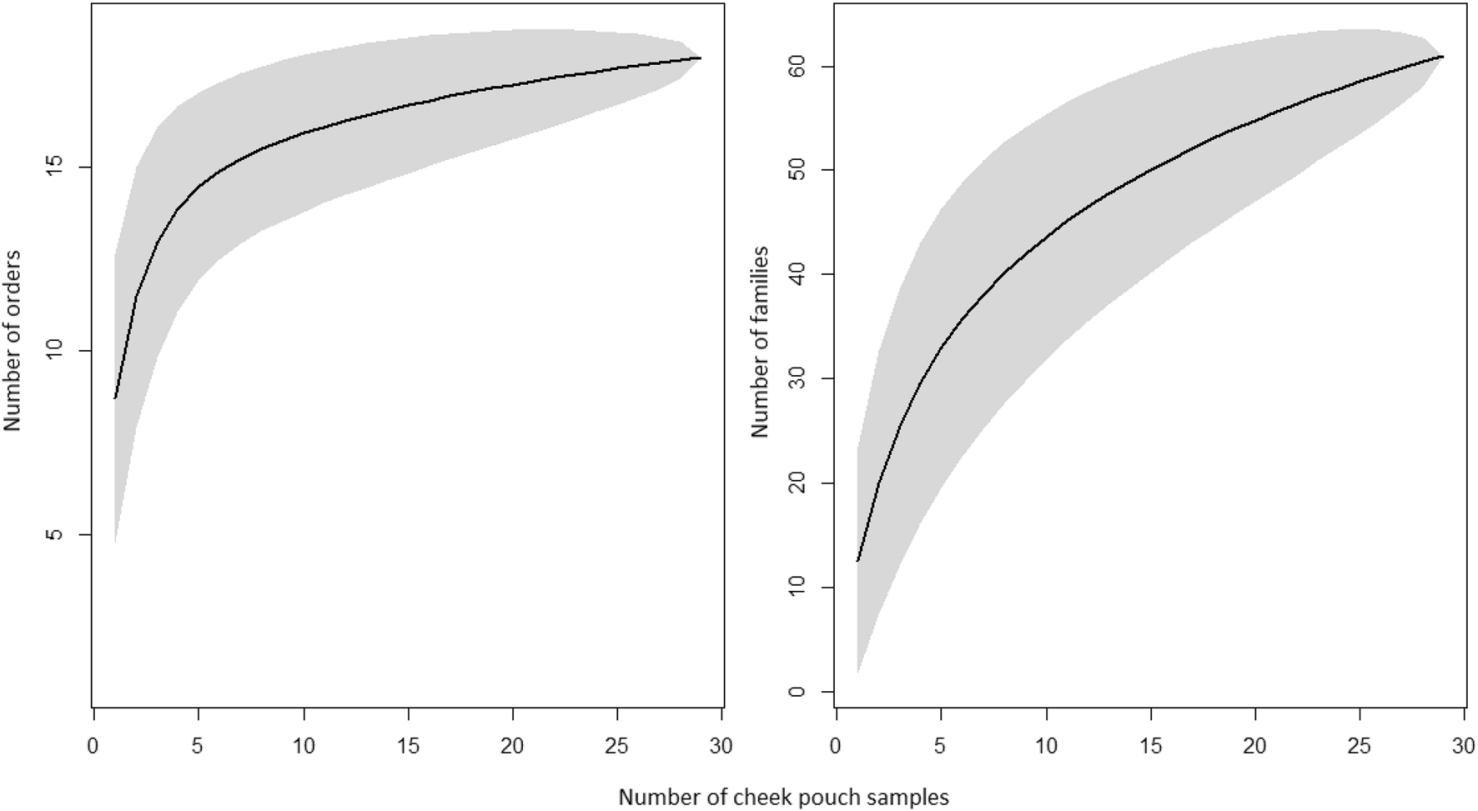
Accumulation curves for (**a**) orders and (**b**) families detected in platypus cheek pouch samples.

We identified a total of 18 orders and 60 families using DNA metabarcoding from 29 collected platypus cheek pouch samples (Appendix 3). Accumulation curves of samples suggest that the more abundant orders and families were detected quickly, within the first to the fifth samples for orders (Fig. 2) but, with more samples, the number of orders and families detected continued to increase.

### Diet

**Figure 3:**
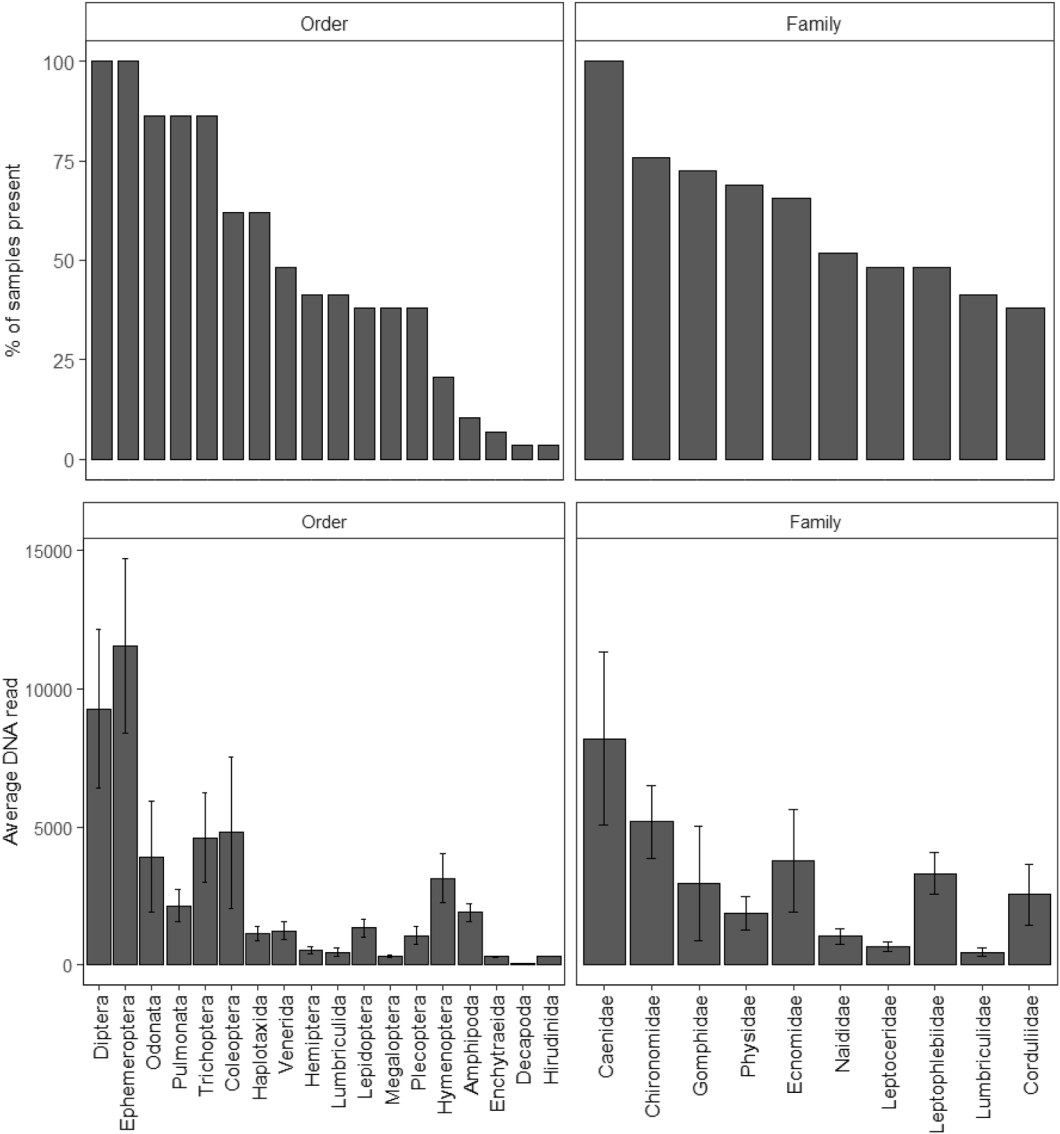
The percentage (%) of samples all orders and the most prevalent 10 families were present in, and the average DNA read of these orders and families from 29 platypus cheek pouch samples.

**Figure 4:**
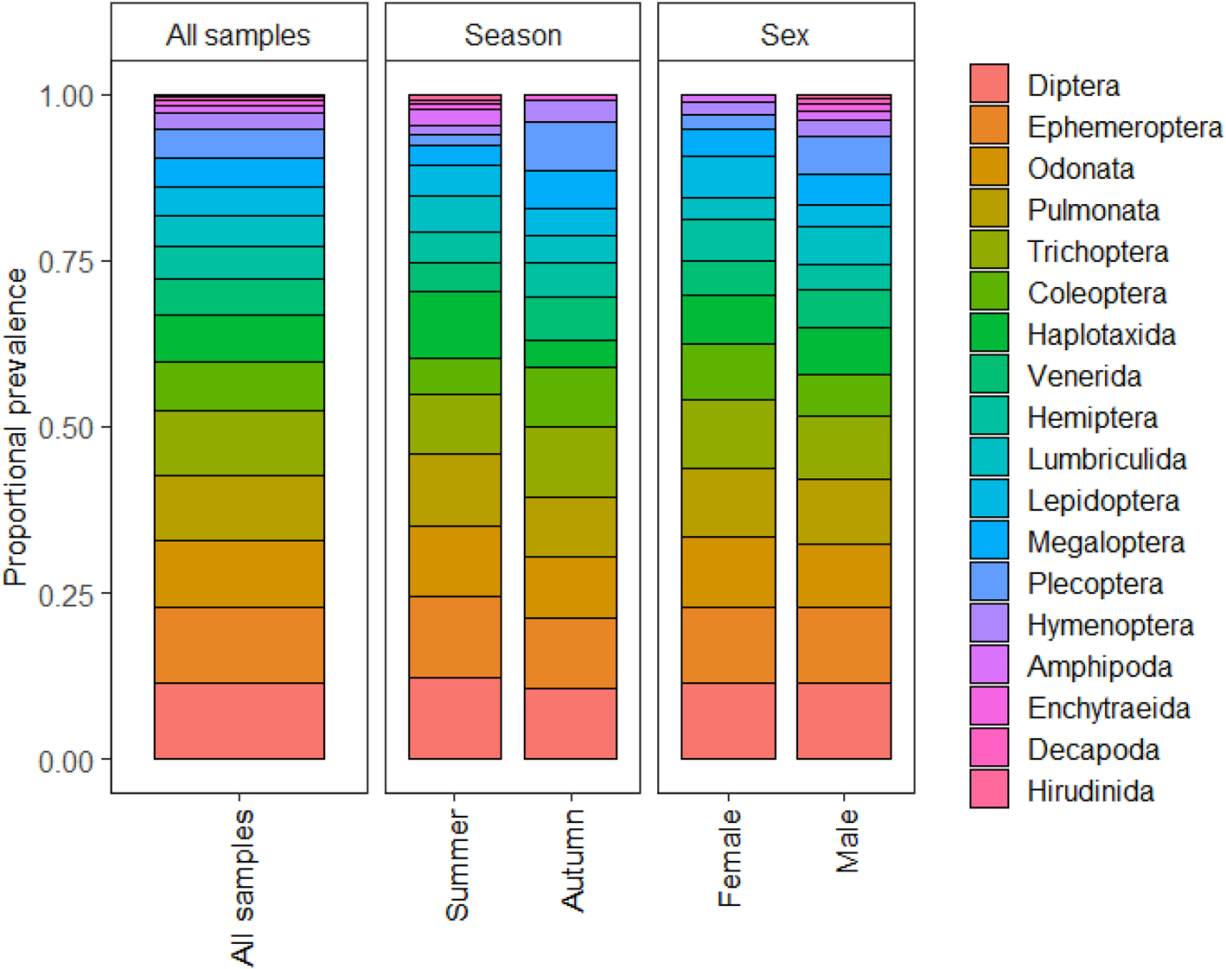
The proportional prevalence of orders across all samples, seasons, and sexes from cheek pouches of 29 platypuses.

The average number of orders detected across all samples was 8.72 ± 0.37 se (range 5–12 orders). Orders Ephemeroptera (mayflies) and Diptera (flies, mosquitos) were most prevalent, both detected in 100% of samples, followed by Trichoptera (caddisflies), Pulmonata (snails), and Odonata (dragonflies, damselflies), all of which were detected in 86.21% of samples (Fig. 3, Appendix 3). Ephemeroptera, Diptera, and Coleoptera had the highest average DNA reads (11,558 ± 3160 se, 9283 ± 2864 se, 4789 ± 2732 se, respectively; Fig. 3). Ephemeroptera and Diptera highest relative prevalence, both making up 11.46% of the diet (Fig. 4).

The average number of families detected across all samples was 12.28 ± 1.01 se (range 3–29). The most prevalent families were Caenidae (mayflies, 100% of samples) and Chironomidae (midges, 75.86%). Caenidae and Chironomidae also had the highest average DNA volume in samples (8195 ± 3129 se, 5188 ± 1331 se, respectively; Fig. 3). Caenidae, Chironomidae, and Gomphidae (dragonflies) had the highest relative prevalence (8.15%, 6.18%, and 5.90%, respectively).

### Seasonal differences

**Figure 5:**
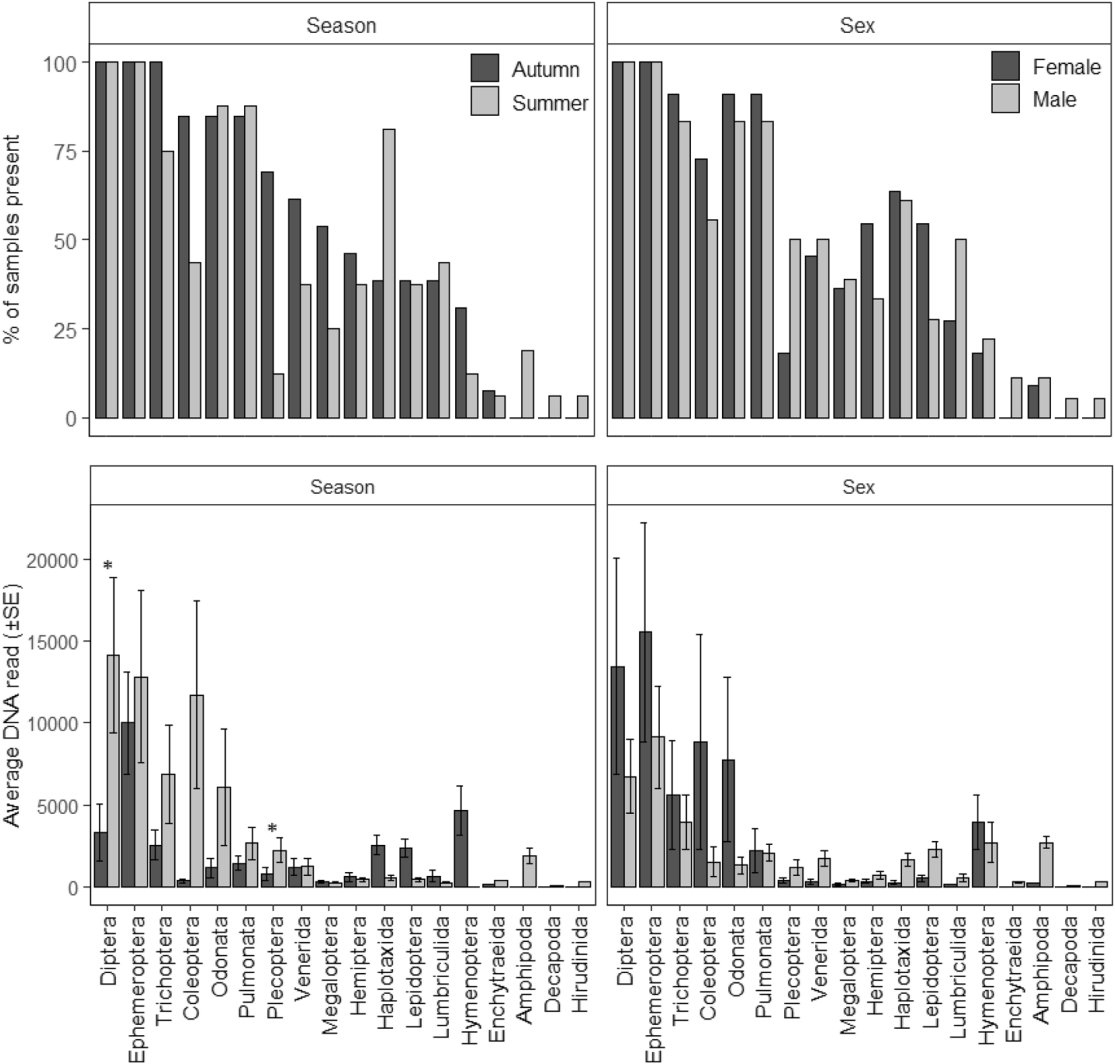
The percentage (%) of samples that orders were present in for seasons and sexes and the average DNA read (± SE) for these orders (* depicts orders significantly different and the P = 0.05 level).

A total of 18 orders and 53 families were detected in samples collected in summer (September-February), compared to 15 orders and 43 families detected from samples collected in autumn (March-August). The average number of orders in the samples was not significantly different between summer (8.19 ± 0.60 se) and autumn (9.38 ± 0.31 se, W = 136.5, P = 0.154). In Summer, orders Diptera and Ephemeroptera were detected in 100% of samples, while in autumn, Diptera, Ephemeroptera, and Trichoptera were all detected in 100% of samples (Fig. 5, Appendix 4). Diptera had the highest average DNA read for summer (14,121 ± 4727) and Ephemeroptera had the highest average DNA read for autumn (10,007 ± 3114 se). DNA reads only differed significantly between seasons for Diptera, higher in summer (W = 58, P = 0.044; Fig. 5) and Plecoptera (stoneflies), higher in autumn (W = 163, P = 0.003). The largest difference in the relative prevalence of orders between seasons was for Plecoptera more prevalent in autumn (7.37% of diet) compared to summer (5.53% of diet; Fig. 4) and Haplotaxida (9.92% of diet in summer, 4.10% in autumn). Orders Amphipoda, Decapoda, and Hirudinida were only detected in summer, although in a small number of samples (Fig. 5).

There was no difference in the number of families detected in samples between summer (11.63 ± 1.59 se) and autumn (13.08 ± 1.18 se, W = 132, P = 0.226). Families Caenidae and Chironomidae were most prevalent during summer (detected in 100% and 87.5% of samples, respectively), while during autumn Caenidae (100%) and Gomphidae (84.62%) were most prevalent. Caenidae had the highest average DNA read within the samples during summer (11,702 ± 5267 se) and Baetidae (mayflies) had the highest for autumn (5780 ± 2363 se). Average DNA reads were significantly higher in summer for Chironomidae (W = 52, P = 0.023). The largest differences in relative prevalence of families between seasons was for Gripopterygidae (stoneflies, 1.08% of diet in summer and 4.71% in autumn).

### Sex differences

Eighteen orders and 53 families were detected in the diet of male platypuses, compared to 15 orders and 44 families in the diet of females. The number of orders detected in the diet of males (8.72 ± 0.51 se) was not significantly different than females (8.73 ± 0.54 se; W = 96, P = 0.909). Diptera and Ephemeroptera were the most prevalent for both sexes, detected in 100% of samples (Appendix 5). Ephemeroptera had the highest average DNA read across samples for both females (15,525 ± 6651 se) and males (9134 ± 3113 se). There was no significant difference in the average DNA reads for any order between males and females (Fig. 5). The relative prevalence of orders between sexes was similar for most orders (Fig. 4).

There was also no difference in the number of families between males (12.72 ± 1.46 se) and females (11.54 ± 1.25 se; W = 99, P = 1.00). Caenidae was the most prevalent family in the diet of both males and females (detected in 100% of samples) and also had the highest average DNA read for females (10,924 ± 6463 se) and males (6527 ± 3247 se). There was no significant difference in the DNA read for any family between males and females (Fig. 5). The relative prevalence of families between sexes was similar for most orders.

## Discussion

This study is the first to use DNA metabarcoding to assess the diet of platypuses, resulting in the detection of 18 orders and 60 families. The most common orders in the diet of the 29 platypus cheek pouch samples we analysed were Ephemeroptera and Diptera, and the most common families were Caenidae and Chironomidae. The number of orders and families in each sample was not significantly affected by season or sex. As in other studies using morphology-based cheek pouch identification and stable isotope analysis of platypus diet (Table 1), the orders Ephemeroptera, Diptera, Trichoptera, and Odonata were all important components of platypus diet, in terms of prevalence and average DNA read^25,27–29^ (Fig. 3).

**Table 1:** The top four (where available) most common and dominant macroinvertebrate orders in the diet of platypuses from studies using different methodologies from different rivers and streams.

Study	River	Method	Most common order (% of samples present/means from mixing model)	Most dominant orders (% of samples dominant)
Faragher^28^	Shoalhaven	Microscopic analysis	Trichoptera (96.7) Diptera (68.9) Coleoptera (62.3) Odonata (52.5)	Trichoptera (52.5) Diptera (14.8) Ephemeroptera (11.5) Odonata (9.8)
Grant^29^	Barnard	Microscopic analysis	Trichoptera (100) Ephemeroptera (75) Coleoptera (66.7) Diptera (50)	Ephemeroptera (50) Trichoptera (41.7) Odonata (8.3)
	Thredbo	Microscopic analysis	Trichoptera (93.8) Ephemeroptera (56.3) Coleoptera (50) Diptera (37.5)	Ephemeroptera (31.3) Trichoptera (12.5)
	Bendora Dam	Microscopic analysis	Trichoptera (71.4) Diptera (57.1)	
	Lake Jindabyne	Microscopic analysis	Ephemeroptera (100) Trichoptera (100) Odonata (60) Hemiptera (60)	Ephemeroptera (60) Trichoptera (20)
McLachlan-Troup et al.^25^	Brogers Creek and Kangaroo River	Microscopic analysis	Trichoptera—leptoceridae (90) Coleoptera—Psephenidae (90) Ephemeroptera—Leptophlebiadae (50) Trichoptera—Helicopsychidae (46)	
Marchant and Grant^30^	Shoalhaven River	Microscopic analysis	Trichoptera (74) Ephemeroptera (61) Odonata (60) Diptera (42)	Trichoptera (32) Odonata (26) Ephemeroptera (16) Diptera (10) Coleoptera (10)
Klamt et al.^27^	Jerrabattgulla Creek and Shoalhaven River	Stable isotope analysis	Diptera/Odonata (0.91 ± 0.07) Trichoptera (0.04 ± 0.05) Hemiptera/ Ephemeroptera/Coleoptera (0.03 ± 0.03)	
This study	Snowy Rivers and Upper Murray Rivers	DNA metabarcoding	Ephemeroptera (100) Diptera (100)	Ephemeroptera Diptera

Previous cheek pouch studies using morphological identification indicate that Diptera were common in the diet of platypuses, but rarely dominant (Table 1), with the conclusion that they are likely avoided because of their small size or inaccessibility^28,29^. Stable isotope and our DNA analyses indicate that they were a dominant component of platypus diet, present in 100% of samples and having the second highest average DNA read across samples (Fig. 3, Appendix 3). This suggests that they may have been underestimated in studies using morphology-based cheek pouch identification, possibly due to increased mastication of these soft-bodied organisms^27,30^. The presence of Diptera in pools where platypuses preferentially forage suggests that they are likely one of the most important dietary components for platypuses^27^. This is supported by the lack of worms in the platypus diet reported from previous studies, despite Marchant and Grant^30^ noting their high abundance in the benthos. Isotope analyses suggested that the combination of Diptera and Odonata were a large dietary component, supporting their importance in the diet, but given they could not be separated isotopically, further investigations, such as our own, are required to establish the importance of each individual order.

Coleoptera was also identified as an important component of platypus diet from other studies (Table 1), but they did not rank as highly from the DNA analyses, being the 6th most prevalent order. Interestingly, the order Pulmonata (snails), equal 3rd most prevalent order, was not identified in previous studies as a common order (Table 1). Previous assessments using morphology-based cheek pouch identification note the difficulties in comparing the finely ground shells of bivalves and gastropods^29^, potentially leading to underestimation in previous studies, with DNA analysis also revealing the order Veneroida (bivalves) were a relatively important dietary component. Reported differences between orders and families in the diet of platypuses between studies may reflect differences in local habitats and macroinvertebrate availability, but may also highlight differences resulting from identification methodology.

While the number of distinct orders and families was higher in summer compared to autumn, there was no significant difference in the average number of orders/families in the samples between these two seasons. Previously reported seasonal differences have been between summer/autumn and winter/spring, when differences in metabolic requirements and prey availability may be greater^25,28^, possibly explaining why we found no differences in the diet when comparing summer and autumn. Only Diptera and Plecoptera had significant differences in the average DNA reads between seasons, possibly reflecting availability.

There have been no reported differences in the diet of male and female platypuses^25,27,28^. DNA analysis suggests males and females had the same dominant orders of Ephemeroptera and Diptera in their diet. McLachlan-Troup et al.^25^ suggest that given males are larger than females, there may be an expected difference in number of taxa consumed between sexes, but that this may not have been detected due to the mastication of prey items in the cheek pouches. However, stable isotope analysis and this DNA analysis also confirms no differences in diet between sexes.

This study was limited by a small sample size and because the samples used in this study were obtained from a number of rivers across two regions (Fig. 1), which may have confounded results given local differences in macroinvertebrate assemblages. Pooling samples into breeding (spring/summer) and non-breeding (winter/autumn) breeding seasons may have also confounded seasonal differences in diet. Future studies can provide greater clarity on dietary selection across seasons by undertaking macroinvertebrate surveys in conjunction with cheek pouch analyses. We also did not undertake morphological analyses of the cheek pouch samples to make comparisons with DNA metabarcoding results. However, given the high sensitivity of this method, the high number of reported orders and families from a relatively small sample size, and similar reported taxa to previous studies, we expect this method would have resulted in the detection of more orders and families than morphological analysis of the cheek pouch samples. The spoon used to collect cheek pouch material was sterilised using 100% ethanol between samples, but was not flame sterilised, which may have contributed to some contamination between samples.

DNA metabarcoding is clearly a useful tool for assessing the diet of platypuses. It improves the ease of identification compared to morphology-based cheek pouch analysis and may increase detection of soft-bodied organisms which may be underestimated using morphology-based methods. While isotopic analysis is also an effective method for assessing platypus diet, variation can occur depending on the part of body sampled, and taxa cannot always be separated isotopically^27^. Future studies should aim to maximise sample sizes to allow for more accurate comparisons between seasons and sexes. There are further opportunities for using DNA metabarcoding for assessing platypus diet. It could be used effectively to compare differences in diet across regions and between rivers, as well as for assessing platypus diet in degraded habitats or downstream of heavily regulated rivers where platypuses are suspected to be declining due to changes in the macroinvertebrate composition^33^.

## Supplementary Information

Supplementary Information.
