# Supplementary material for: Using DNA metabarcoding as a novel approach for analysis of platypus diet
Source: Sci Rep. 2022 Feb 10;12:2247. doi: 10.1038/s41598-022-06023-y (PMC8831530; doi:10.1038/s41598-022-06023-y)
Supplement: Supplementary file 1 — Supplementary Information. [file 41598_2022_6023_MOESM1_ESM.docx]

# Appendix

## Appendix 1

Table A1. Details on the catchment, river (see Figure 1), capture date, season, weight, length, sex, and ages of platypuses that cheek pouch samples were obtained from.

| **Platypus** | **Catchment** | **River** | **Date** | **Month** | **Season** | **Weight** | **Length** | **Sex** | **Age** |
| --- | --- | --- | --- | --- | --- | --- | --- | --- | --- |
| E3 | Snowy Rivers | Eucumbene DS | 12/01/2016 | 12 | Summer | 0.87 | 455 | F | A |
| E2 |  |  | 12/02/2016 | 12 | Summer | 0.89 | 420 | F | A |
| E5 |  |  | 12/05/2016 | 12 | Summer | 1.58 | 540 | M | A |
| E65 |  | Eucumbene US | 3/17/2017 | 3 | Autumn | 0.58 | 405 | F | J |
| E69 |  |  | 3/28/2017 | 3 | Autumn | 0.56 | 364 | F | J |
| E70 |  |  | 3/29/2017 | 3 | Autumn | 1.36 | 497 | M | A |
| E41 |  | Snowy | 2/23/2017 | 2 | Summer | 0.52 | 390 | M | J |
| E42 |  |  | 2/25/2017 | 2 | Summer | 0.47 | 360 | M | J |
| E46 |  |  | 2/25/2017 | 2 | Summer | 0.86 | 415 | F | A |
| E48 |  |  | 2/27/2017 | 2 | Summer | 0.89 | 445 | F | A |
| E53 |  |  | 3/01/2017 | 3 | Autumn | 0.89 | 428 | F | A |
| E67 |  |  | 3/19/2017 | 3 | Autumn | 1.47 | 520 | M | A |
| E71 |  |  | 4/10/2017 | 4 | Autumn | 1.59 | 535 | M | A |
| E84 |  |  | 4/25/2017 | 4 | Autumn | 1.53 | 515 | M | A |
| E85 |  |  | 4/26/2017 | 4 | Autumn | 1.67 | 553 | M | A |
| E93 |  |  | 8/23/2017 | 8 | Autumn | 0.64 | 413 | M | SA |
| E98 |  |  | 8/27/2017 | 8 | Autumn | 1.15 | 480 | M | A |
| E101 |  |  | 9/24/2017 | 9 | Summer | 1.66 | 540 | M | A |
| E104 |  |  | 9/25/2017 | 9 | Summer | 1.23 | 510 | M | A |
| E109 |  |  | 10/28/2017 | 10 | Summer | 1.55 | 535 | M | A |
| E38 |  |  | 2/21/2017 | 2 | Summer | 1.52 | 525 | M | A |
| E37 |  |  | 2/22/2017 | 2 | Summer | 1.29 | 490 | M | A |
| E61 |  | Thredbo | 3/15/2017 | 3 | Autumn | 0.9 | 472 | M | A |
| V36 | Upper Murray Rivers | Mitta Mitta DS | 5/13/2018 | 5 | Autumn | 1.64 | 554 | M | A |
| V23 |  |  | 3/07/2018 | 3 | Autumn | 1.18 | 490 | M | A |
| V25 |  | Mitta Mitta US | 3/08/2018 | 3 | Autumn | 0.51 | 370 | F | J |
| V31 |  | Mitta Mitta US | 5/03/2018 | 5 | Autumn | 0.46 | 377 | F | J |
| V6 |  | Ovens | 2/03/2018 | 2 | Summer |  | 410 | F | A |
| V7 |  |  | 2/04/2018 | 2 | Summer |  | 440 | F | A |

## Appendix 2

Table A2. Taxonomic groups removed prior to analysis because they were considered environmental DNA, parasitic organisms, or non-target, small organisms consumed incidentally by platypuses.

| **Kingdoms** | Alveolata |
| --- | --- |
|  | Excavata |
|  | Fungi |
|  | Hacrobia |
|  | Plantae |
|  | Protozoa |
|  | Rhizaria |
|  | Stramenopiles |
| **Phyla** | Chordata |
|  | Echinodermata |
|  | Gastrotricha |
|  | Nematoda |
|  | Nematomorpha |
|  | Nemertea |
|  | Placozoa |
|  | Porifera |
|  | Rotifera |
| **Classes** | Trematoda |
|  | Ostracoda |
|  | Hexanauplia |
|  | Branchiopoda |
|  | Cephalopoda |
|  | Arachnida |
|  | Diplopoda |
|  | Pauropoda |
|  | Turbellaria |
| **Orders** | Isopoda |
|  | Orthoptera |
|  | Thysanoptera |
|  | Trombidiformes |
|  | Protura |

## Appendix 3

Table A3. The percentage of platypus cheek pouch samples which each taxa were present in, and the average DNA read (±standard error) of each taxa across samples.

| **Order**  **(common name)** | **% samples present** | **Average DNA read±SE** | **Family** | **% samples present** | **Average DNA read±SE** |
| --- | --- | --- | --- | --- | --- |
| Diptera (flies) | 100 | 9283±2864 | Chironomidae | 75.86 | 5188±1331 |
|  |  |  | Simuliidae | 24.14 | 829±184 |
|  |  |  | Limoniidae | 13.79 | 311±68 |
|  |  |  | Tipulidae | 10.34 | 145±5 |
|  |  |  | Chloropidae | 6.90 | 242±43 |
|  |  |  | Drosophilidae | 3.45 | 17 |
|  |  |  | Empididae | 3.45 | 229 |
|  |  |  | Mycetophilidae | 3.45 | 14 |
| Ephemeroptera (mayflies) | 100 | 11558±3160 | Caenidae | 100.00 | 8195±3129 |
|  |  |  | Leptophlebiidae | 48.28 | 3317±778 |
|  |  |  | Baetidae | 24.14 | 2538±1073 |
|  |  |  | Coloburiscidae | 24.14 | 887±177 |
| Odonata (damselflies, dragonflies) | 86.21 | 3909±2012 | Gomphidae | 72.41 | 2946±2093 |
|  |  |  | Corduliidae | 37.93 | 2551±1109 |
|  |  |  | Coenagrionidae | 20.69 | 428±149 |
|  |  |  | Argiolestidae | 10.34 | 734±117 |
|  |  |  | Calopterygidae | 6.90 | 69±4 |
|  |  |  | Aeshnidae | 3.45 | 99 |
|  |  |  | Synlestidae | 3.45 | 13 |
| Pulmonata (snails) | 86.21 | 2134±588 | Physidae | 68.97 | 1869±585 |
|  |  |  | Lymnaeidae | 31.03 | 996±425 |
|  |  |  | Ancylidae | 17.24 | 389±77 |
|  |  |  | Agriolimacidae | 3.45 | 13 |
| Trichoptera (caddisflies) | 86.21 | 4606±1619 | Ecnomidae | 65.52 | 3769±1850 |
|  |  |  | Leptoceridae | 48.28 | 645±180 |
|  |  |  | Conoesucidae | 24.14 | 263±27 |
|  |  |  | Hydrobiosidae | 24.14 | 1474±350 |
|  |  |  | Hydropsychidae | 20.69 | 318±38 |
|  |  |  | Hydroptilidae | 13.79 | 87±12 |
|  |  |  | Polycentropodidae | 13.79 | 490±99 |
|  |  |  | Philorheithridae | 10.34 | 224±36 |
|  |  |  | Helicopsychidae | 6.90 | 577±135 |
|  |  |  | Philopotamidae | 6.90 | 104±24 |
|  |  |  | Calamoceratidae | 6.90 | 638±141 |
|  |  |  | Limnephilidae | 3.45 | 568 |
|  |  |  | Glossosomatidae | 3.45 | 19 |
| Coleoptera (beetles) | 62.07 | 4789±2732 | Elmidae | 6.90 | 317±78 |
|  |  |  | Scirtidae | 6.90 | 656±48 |
|  |  |  | Carabidae | 3.45 | 592 |
|  |  |  | Dytiscidae | 3.45 | 280 |
|  |  |  | Hydrophilidae | 3.45 | 18 |
| Haplotaxida (worms) | 62.07 | 1126±272 | Naididae | 51.72 | 1034±288 |
|  |  |  | Lumbricidae | 24.14 | 670±148 |
| Venerida (bivalves) | 48.28 | 1235±327 | Sphaeriidae | 34.48 | 992±289 |
|  |  |  | Corbiculidae | 20.69 | 1230±359 |
| Hemiptera (true bugs) | 41.38 | 532±123 | Micronectidae | 31.03 | 596±135 |
|  |  |  | Corixidae | 10.34 | 277±65 |
|  |  |  | Lachnidae | 6.90 | 51±11 |
| Lumbriculida (worms) | 41.37 | 447±157 | Lumbriculidae | 41.38 | 447±157 |
| Lepidoptera (butterflies, moths) | 37.93 | 1334±312 | Noctuidae | 6.90 | 1984±517 |
|  |  |  | Lycaenidae | 3.45 | 5032 |
| Megaloptera (alderflies, dobsonflies) | 37.96 | 314±46 | Corydalidae | 31.03 | 256±44 |
|  |  |  | Sialidae | 3.45 | 544 |
| Plecoptera (stoneflies) | 37.93 | 1053±319 | Gripopterygidae | 34.48 | 1088±335 |
|  |  |  | Eustheniidae | 3.45 | 684 |
| Hymenoptera (sawflies, wasps, bees, ants) | 20.69 | 3129±885 | Braconidae | 3.45 | 58 |
|  |  |  | Formicidae | 3.45 | 55 |
| Amphipoda (crustaceans) | 10.34 | 1900±327 | Chiltoniidae | 10.34 | 1900±327 |
| Enchytraeida (worms) | 6.90 | 289±37 |  |  |  |
| Decapoda (crustaceans) | 3.45 | 67±0 | Atyidae | 3.45 | 67 |
| Hirudinida (leeches) | 3.45 | 307±0 |  |  |  |
| No order |  |  | Capitellidae | 17.24 | 175±23 |

## Appendix 4

Table A4. The percentage of platypus cheek pouch samples which each taxa were present in, and the average DNA read (±standard error) of each taxa across samples for summer and autumn.

| **Order (common name)** | **% samples present summer/autumn** | **Average DNA read±SE summer/autumn** | **Family** | **% samples present summer/autumn** | **Average DNA read±SE summer/autumn** |
| --- | --- | --- | --- | --- | --- |
| Diptera (flies) | 100/100 | 14121±4727/ 3328±1723 | Chironomidae | 87.5/61.54 | 7276±2073/ 1533±443 |
|  |  |  | Simuliidae | 31.25/15.38 | 546±164/ 1538±462 |
|  |  |  | Limoniidae | 18.75/7.69 | 298±112/ 348 |
|  |  |  | Tipulidae | 12.5/7.69 | 149±9/ 137 |
|  |  |  | Chloropidae | 12.5/0 | 242±57/0 |
|  |  |  | Drosophilidae | 6.25/0 | 17/0 |
|  |  |  | Empididae | 6.25/0 | 229/0 |
|  |  |  | Mycetophilidae | 6.25/0 | 14/0 |
| Ephemeroptera (mayflies) | 100/100 | 12819±5221/ 10007±3114 | Caenidae | 100/100 | 11702±5267/ 3878±2341 |
|  |  |  | Leptophlebiidae | 37.5/61.54 | 2070±977/ 4253±1220 |
|  |  |  | Baetidae | 25/23.08 | 107±37/ 5780±2363 |
|  |  |  | Coloburiscidae | 18.75/30.77 | 741±243/ 997±298 |
| Odonata (damselflies, dragonflies) | 87.50/84.62 | 6067±3552/ 1164±591 | Gomphidae | 62.5/84.62 | 5227±4090/ 872±501 |
|  |  |  | Corduliidae | 43.75/30.77 | 3881±1833/ 222±65 |
|  |  |  | Coenagrionidae | 25/15.38 | 578±247/ 130±48 |
|  |  |  | Argiolestidae | 12.5/7.69 | 483±160/1238 |
|  |  |  | Calopterygidae | 0/15.38 | 0/69±6 |
|  |  |  | Aeshnidae | 0/7.69 | 0/99 |
|  |  |  | Synlestidae | 6.25/0 | 13/0 |
| Pulmonata (snails) | 87.50/84.62 | 2661±990/ 1464±465 | Physidae | 68.75/69.23 | 2588±1015/ 990±328 |
|  |  |  | Lymnaeidae | 37.5/23.08 | 1190±701/ 609±278 |
|  |  |  | Ancylidae | 12.5/23.08 | 802/595±118 |
|  |  |  | Agriolimacidae | 6.25/0 | 13/0 |
| Trichoptera (caddisflies) | 75.00/100.00 | 6844±2996/ 2541±906 | Ecnomidae | 56.25/76.92 | 7225±3503/ 658±303 |
|  |  |  | Leptoceridae | 43.75/53.85 | 405±171/ 885±332 |
|  |  |  | Conoesucidae | 12.5/38.46 | 121±16/ 319±37 |
|  |  |  | Hydrobiosidae | 6.25/46.15 | 61/1710±540 |
|  |  |  | Hydropsychidae | 12.5/30.77 | 297±85/ 329±49 |
|  |  |  | Hydroptilidae | 6.25/23.08 | 75/91±22 |
|  |  |  | Polycentropodidae | 6.25/23.08 | 99/620±158 |
|  |  |  | Philorheithridae | 6.25/15.38 | 212/230±76 |
|  |  |  | Helicopsychidae | 0/15.38 | 0/577±202 |
|  |  |  | Philopotamidae | 6.25/7.69 | 6.25/13 |
|  |  |  | Calamoceratidae | 0/15.38 | 0/638±211 |
|  |  |  | Limnephilidae | 6.25/0 | 568/0 |
|  |  |  | Glossosomatidae | 6.25/0 | 19/0 |
| Coleoptera (beetles) | 43.75/84.62 | 11706±5711/ 387±109 | Elmidae | 6.25/7.69 | 614/614 |
|  |  |  | Scirtidae | 12.5/0 | 656±65/0 |
|  |  |  | Carabidae | 6.25/0 | 592/0 |
|  |  |  | Dytiscidae | 6.25/0 | 280/0 |
|  |  |  | Hydrophilidae | 6.25/0 | 18/0 |
| Haplotaxida (worms) | 81.25/38.46 | 570±163/  2570±571 | Naididae | 62.5/38.46 | 356±99/ 2391±594 |
|  |  |  | Lumbricidae | 31.25/15.38 | 759±235/ 448±102 |
| Venerida (bivalves) | 37.50/61.54 | 1244±487/  1229±484 | Sphaeriidae | 18.75/53.85 | 504±139/ 1201±508 |
|  |  |  | Corbiculidae | 18.75/23.08 | 1984±686/ 476±97 |
| Hemiptera (true bugs) | 37.50/46.15 | 450±89/ 614±252 | Micronectidae | 25/38.46 | 456±89/ 707±267 |
|  |  |  | Corixidae | 18.75/0 | 277±87/0 |
|  |  |  | Lachnidae | 6.25/7.69 | 9/93 |
| Lumbriculida (worms) | 43.75/38.46 | 285±75/ 675±365 | Lumbriculidae | 43.75/38.46 | 285±75/ 675±365 |
| Lepidoptera (butterflies, moths) | 37.50/38.46 | 451±140/ 2395±561 | Noctuidae | 6.25/7.69 | 17/3951 |
|  |  |  | Lycaenidae | 0/7.69 | 0/5032 |
| Megaloptera (alderflies, dobsonflies) | 25.00/53.85 | 285±64/ 331±73 | Corydalidae | 18.75/46.15 | 198±58/ 285±72 |
|  |  |  | Sialidae | 6.25/0 | 544/0 |
| Plecoptera (stoneflies) | 12.50/69.23 | 2256±785/ 785±393 | Gripopterygidae | 12.5/61.54 | 2256±785/ 796±420 |
|  |  |  | Eustheniidae | 0/7.69 | 0/684 |
| Hymenoptera (sawflies, wasps, bees, ants) | 12.50/30.77 | 34±9/ 4677±1476 | Braconidae | 6.25/0 | 58/0 |
|  |  |  | Formicidae | 0/7.69 | 0/55 |
| Amphipoda (crustaceans) | 18.75/0 | 1900±441/0 | Chiltoniidae | 18.75/0 | 1900±441/0 |
| Enchytraeida (worms) | 6.25/7.69 | 431/147 |  |  |  |
| Decapoda (crustaceans) | 6.25/  0.00 | 67/0 | Atyidae | 6.25/0 | 67/0 |
| Hirudinida (leeches) | 6.25/0 | 307/0 |  |  |  |
| No order |  |  | Capitellidae | 18.75/15.38 | 141±35/  227±35 |

## Appendix 5

Table A5. The percentage of platypus cheek pouch samples which each taxa were present in, and the average DNA read (±standard error) of each taxa across samples for males and females.

| **Order (common name)** | **% samples present female/male** | **Average DNA read±SE female/male** | **Family** | **% samples present female/male** | **Average DNA read±SE female/male** |
| --- | --- | --- | --- | --- | --- |
| Diptera (flies) | 100/100 | 13443±6594/ 6741±2267 | Chironomidae | 63.64/83.33 | 8300±2945/ 3735±1271 |
|  |  |  | Simuliidae | 18.18/27.78 | 105±8/ 1119±248 |
|  |  |  | Limoniidae | 9.09/16.67 | 59/395±95 |
|  |  |  | Tipulidae | 0/16.67 | 0/145±6 |
|  |  |  | Chloropidae | 9.09/5.56 | 404/80 |
|  |  |  | Drosophilidae | 0/5.56 | 0/17 |
|  |  |  | Empididae | 0/5.56 | 0/229 |
|  |  |  | Mycetophilidae | 0/5.56 | 0/14 |
| Ephemeroptera (mayflies) | 100/100 | 15525±6651/ 9134±3113 | Caenidae | 100/100 | 10924±6463/ 6527±3247 |
|  |  |  | Leptophlebiidae | 54.55/44.44 | 3710±1173/ 3023±1094 |
|  |  |  | Baetidae | 27.27/22.22 | 5710±2590/ 159±36 |
|  |  |  | Coloburiscidae | 36.36/16.67 | 1066±343/ 648±189 |
| Odonata (damselflies, dragonflies) | 90.91/83.33 | 7769±5030/ 1336±504 | Gomphidae | 81.82/66.67 | 5956±5184/ 689±406 |
|  |  |  | Corduliidae | 36.36/38.89 | 5437±2983/ 901±317 |
|  |  |  | Coenagrionidae | 0/33.33 | 0/428±190 |
|  |  |  | Argiolestidae | 18.18/5.56 | 1087±64/29 |
|  |  |  | Calopterygidae | 9.09/5.56 | 52/85 |
|  |  |  | Aeshnidae | 9.09/0 | 99/0 |
|  |  |  | Synlestidae | 0/5.56 | 0/13 |
| Pulmonata (snails) | 90.91/83.33 | 2216±1314/ 2080±526 | Physidae | 72.73/66.67 | 2501±1468/ 1447±295 |
|  |  |  | Lymnaeidae | 18.18/38.89 | 12±2/ 1278±605 |
|  |  |  | Ancylidae | 27.27/11.11 | 567±141/ 122±13 |
|  |  |  | Agriolimacidae | 0/5.56 | 0/13 |
| Trichoptera (caddisflies) | 90.91/83.33 | 5604±3343/ 3941±1667 | Ecnomidae | 72.73/61.11 | 4719±3857/ 3078±1868 |
|  |  |  | Leptoceridae | 45.45/50.00 | 881±448/ 514±143 |
|  |  |  | Conoesucidae | 18.18/27.78 | 195±50/ 290±36 |
|  |  |  | Hydrobiosidae | 27.27/22.22 | 1516±257/ 1443±606 |
|  |  |  | Hydropsychidae | 27.27/16.67 | 318±58/ 319±62 |
|  |  |  | Hydroptilidae | 9.09/16.67 | 66/94±18 |
|  |  |  | Polycentropodidae | 9.09/16.67 | 76/628±132 |
|  |  |  | Philorheithridae | 9.09/11.11 | 37/318±35 |
|  |  |  | Helicopsychidae | 9.09/5.56 | 61/1092 |
|  |  |  | Philopotamidae | 0/11.11 | 0/104±30 |
|  |  |  | Calamoceratidae | 9.09/5.56 | 1175/101 |
|  |  |  | Limnephilidae | 0/5.56 | 0/568 |
|  |  |  | Glossosomatidae | 9.09/0 | 19/0 |
| Coleoptera (beetles) | 72.73/55.56 | 8850±6548/ 1540±937 | Elmidae | 18.18/0 | 317±127/0 |
|  |  |  | Scirtidae | 9.09/5.56 | 840/472 |
|  |  |  | Carabidae | 9.09/0 | 592/0 |
|  |  |  | Dytiscidae | 0/5.56 | 0/280 |
|  |  |  | Hydrophilidae | 0/5.56 | 0/18 |
| Haplotaxida (worms) | 63.64/61.11 | 279±140/  1664±387 | Naididae | 54.55/50.00 | 324±149/ 1508±435 |
|  |  |  | Lumbricidae | 9.09/33.33 | 15/780±192 |
| Venerida (bivalves) | 45.45/50.00 | 339±160/  1733±478 | Sphaeriidae | 36.36/33.33 | 422±174/ 1372±455 |
|  |  |  | Corbiculidae | 9.09/27.78 | 9/1474±485 |
| Hemiptera (true bugs) | 54.55/33.33 | 341±120/  722±200 | Micronectidae | 27.27/33.33 | 405±130/ 691±202 |
|  |  |  | Corixidae | 9.09/11.11 | 677/78±14 |
|  |  |  | Lachnidae | 18.18/0 | 51±18/0 |
| Lumbriculida (worms) | 27.27/50.00 | 177±27/  537±230 | Lumbriculidae | 27.27/50.00 | 177±27/ 537±230 |
| Lepidoptera (butterflies, moths) | 54.55/27.78 | 533±184/  2296±499 | Noctuidae | 0/11.11 | 0/1984±656 |
|  |  |  | Lycaenidae | 0/5.56 | 0/5032 |
| Megaloptera (alderflies, dobsonflies) | 36.36/38.89 | 188±66/  387±59 | Corydalidae | 36.36/27.78 | 188±66/ 311±62 |
|  |  |  | Sialidae | 0/5.56 | 0/544 |
| Plecoptera (stoneflies) | 18.18/50.00 | 411±157/  1195±442 | Gripopterygidae | 18.18/44.44 | 411±157/ 1257±470 |
|  |  |  | Eustheniidae | 0/5.56 | 0/684 |
| Hymenoptera (sawflies, wasps, bees, ants) | 18.18/22.22 | 3969±1668/ 2709±1225 | Braconidae | 9.09/0 | 58/0 |
|  |  |  | Formicidae | 9.09/0 | 55/0 |
| Amphipoda (crustaceans) | 9.09/11.11 | 258/2722±348 | Chiltoniidae | 9.09/11.11 | 258/2722±348 |
| Enchytraeida (worms) | 0/11.11 | 0/289±47 |  |  |  |
| Decapoda (crustaceans) | 0/5.56 | 0/67 | Atyidae | 0/5.56 | 0/5.56 |
| Hirudinida (leeches) | 0/5.56 | 0/307 |  |  |  |
| No order |  |  | Capitellidae | 9.09/22.22 | 137/185±34 |
